# Preconception Health Research Priorities for Adolescents and Young Adults in Australia

**DOI:** 10.1111/hex.70950

**Published:** 2026-10-28

**Authors:** Zahra Ali Padhani, Jodie C. Avery, Gizachew A. Tessema, Keeth L. B. Mayakaduwage, Jacqueline A. Boyle, Danielle Mazza, Sara Ataie, Salima Meherali, Zohra S. Lassi

**Affiliations:** ^1^ School of Public Health, College of Health, Adelaide University Adelaide South Australia Australia; ^2^ Robinson Research Institute, Adelaide University Adelaide South Australia Australia; ^3^ Curtin School of Population Health, Curtin University Perth Western Australia Australia; ^4^ Department of Obstetrics and Gynaecology, School of Clinical Sciences at Monash Health, Faculty of Medicine, Nursing and Health Sciences Monash University Melbourne Victoria Australia; ^5^ Monash Women's, Monash Health Melbourne Victoria Australia; ^6^ Eastern Health Clinical School, Faculty of Medicine, Nursing and Health Sciences Monash University Melbourne Victoria Australia; ^7^ Department of General Practice, School of Public Health and Preventive Medicine, Faculty of Medicine Nursing and Health Sciences Monash University Melbourne Victoria Australia; ^8^ College of Medicine and Public Health Flinders University Adelaide South Australia Australia; ^9^ College of Health Sciences, Faculty of Nursing, Edmonton Clinic Health Academy, University of Alberta Edmonton Alberta Canada

**Keywords:** adolescents, Australia, consumer involvement, interest‐holders, preconception, research priority, young adults

## Abstract

**Background:**

Guidelines on pre‐pregnancy counselling are primarily clinical, and although recommendations and policy documents on preconception care exist in Australia, they place little or no emphasis on the preconception health of adolescents and young adults.

**Objective:**

To identify and prioritise unanswered questions and evidence uncertainties concerning preconception health needs of adolescents and young adults residing in Australia.

**Design:**

Research priority exercise.

**Setting and Participants:**

Participants included young interest‐holders (18–24 years) and professional interest‐holders from the academic, healthcare, policy, community and government sectors residing in Australia.

**Methods:**

James Lind Alliance (JLA) methodology was followed to identify research priorities for preconception health of adolescents and young adults. The process was led by a multidisciplinary steering committee comprising young interest‐holders and professional interest‐holders (including academics and clinicians). A rapid literature review was conducted, from which 80 research questions were developed across 10 domains, which were refined through consultation and prioritised via two rounds of online surveys on Qualtrics using a 9‐point Likert scale.

**Results:**

The participants included 14 young interest‐holders in each survey round, with 22 professional interest‐holders in the first round and 30 in the second. Participants from across Australia participated in the survey, but most were from South Australia. In the first survey round, 28 questions across seven domains were prioritised by both professional and young interest‐holders. This was followed by a reprioritisation exercise, resulting in the final top 10 research questions spanning five domains. The highest‐priority research questions identified by the interest‐holders concentrated in the domains of violence and mental health; early intervention and prevention; smoking, tobacco, alcohol, and substance use; access to preconception care and the healthcare system; and priority populations.

**Conclusion:**

The study identified the top 10 priority research questions informed by professional and young interest‐holders. It promotes new research and collaboration while offering guidance on future research investments and on designing preconception interventions for adolescents and young adults in Australia. Turning these priorities into research could improve the health outcomes for adolescents and their future generations.

## 1 Introduction

Despite recent advances, increasing prenatal care services and research, many individuals are not healthy at the time of conception [1]. Health prior to conception is associated with maternal, perinatal and neonatal health outcomes [1]. Preconception, defined as the period before pregnancy or during active pregnancy planning, offers a window of improvement when individuals of reproductive age have an opportunity to improve their health and health‐related behaviours [2]. Preconception care involves counselling and social, behavioural and biomedical interventions for women and their partners before pregnancy to improve health outcomes [1]. Behavioural modifications before pregnancy, such as weight management and lifestyle changes (e.g., increasing physical activity, avoiding alcohol, drug and tobacco use), can be positive steps to improve the subsequent health of the pregnant person and baby [3]. Optimal health prior to conception can help reduce the risk of anaemia, preterm birth, stillbirth, infertility, congenital anomaly and maternal and child mortality [4]. Among adolescents and young adults, preconception care extends beyond pregnancy planning and includes improving health literacy, promoting healthy behaviours, and building lifestyle capacities to prevent health risk factors and improve health outcomes [5, 6].

According to a recent study, approximately one in four women experiences an unintended pregnancy, with around one‐third ending in abortion in Australia [7]. These pregnancies are often associated with social and structural disadvantage, including younger age, exposure to abuse, poverty, unemployment, lower educational attainment, limited social support and health system barriers [7, 8]. In 2023, the age‐specific fertility rate among adolescents aged 15–19 years was 6.2 births per 1000 women and 32.9 births per 1000 women among young adults aged 20–24 years [9]. The fertility rates among younger women highlight the importance of strengthening preconception health during adolescence. The findings from the Australian Contraceptive ChOice pRoject (ACCORd) trial suggest improved contraception counselling and access to long‐acting reversible contraceptives to reduce unintended pregnancies and abortion rates [7, 10]. Its findings also highlight the need for targeted preconception interventions beyond contraception, including health literacy, lifestyle modification and access to care.

Preconception knowledge also plays a critical role in shaping health behaviours and attitudes towards pregnancy planning [11]. Increasingly, pregnancy planning is recognised not as a discrete pre‐pregnancy event, but as a continuum extending across the life course [12]. Recent evidence suggests substantial gaps in knowledge of preconception health and fertility among women, including men and adolescents [13, 14, 15]. A major obstacle to using preconception care is the lack of awareness of preconception risk factors [14]. These gaps are concerning because both men and women share the endeavour of childbearing, and men's health, such as sperm quality and genetics, affects pregnancy and neonatal health outcomes [16]. Second, the World Health Organization (WHO) has identified adolescents (10–19 years) as a vulnerable population that has received insufficient targeted attention [17]. They are more prone to be exposed to risk factors, while also facing barriers to accessing care, engaging in preventive health behaviours and leaving unsafe environments [18, 19]. Risk‐related behaviours during adolescence, including violence, poor diet, substance use, smoking and unprotected sexual activity, can have significant consequences for both immediate health and future reproductive outcomes [18, 20, 21].

Several preconception health frameworks, recommendations and guidelines exist in Australia. However, their implementation remains limited. Clinical guidelines for pre‐pregnancy counselling are available for healthcare practitioners, which primarily focus on individuals who are actively planning a pregnancy. Currently, there are no specific guidelines or targeted strategies for promoting and delivering preconception health interventions among adolescents and young adults who are not planning pregnancy, despite the importance of supporting this population to prepare for future reproductive health and to maintain good health throughout the life course [22]. Adolescents and young adults also encounter barriers to accessing preconception care, which remains a major barrier. Additional challenges include lack of awareness, issues with affordability and confidentiality, and financial limitations [23, 24]. According to the existing evidence, Australian people of reproductive age (both women and their partners) are willing and eager to learn about preconception care to adopt healthy behaviours [24]. A qualitative study found that while adolescents considered starting a family a far‐distant notion, they expressed interest in gaining more information on preconception health and fertility [13].

Evidence suggests that promotion of preconception care can improve health behaviours, including diet, physical activity and smoking cessation, while also enhancing self‐efficacy and knowledge and reducing risk factors, among both men and women, particularly those with chronic medical conditions [25]. However, given the broad scope of preconception health and limited evidence specific to adolescents and young adults, important research gaps remain. There is a need to identify research uncertainties in this area to support a preventive approach that promotes healthier pregnancies, improves the identification and management of risk factors and informs the development of interventions that can be delivered across diverse settings, including schools, health services and community contexts, ultimately improving outcomes for families.

## 2 Objective

This study aimed to identify and prioritise unanswered questions and evidence uncertainties concerning the preconception health needs of adolescents and young adults residing in Australia.

## 3 Methods

A research priority‐setting exercise was conducted following James Lind Alliance (JLA) methodology [26]. JLA provides a guide for establishing and managing the Priority Setting Partnership (PSP), bringing stakeholders/topic experts and consumers together to identify and prioritise unanswered questions or evidence uncertainties. The JLA‐PSP process helps shortlist the top 10 priorities, highlighting important areas of research. These are broad, important areas prioritised by stakeholders and consumers for further research. The Guidance for Reporting Involvement of Patients and the Public (GRIPP 2) checklist was used for reporting results (Table S1).

In this study, stakeholders and consumers were referred to as interest‐holders. Healthcare professionals, academics, clinicians and policymakers were referred to as professional interest‐holders, whereas adolescents and young adults were referred to as young interest‐holders. The term interest‐holders was recommended by the Multi‐Stakeholder Engagement Consortium (MuSE) to replace the term ‘stakeholders’ [27]. It is defined as ‘groups with legitimate interests in the health issue under consideration’ [27]. The term ‘stakeholders’ was replaced to avoid colonial connotations linked to ‘staking a claim’ on occupied or stolen property from Indigenous People.

Following the JLA methodology, the following steps were undertaken to conduct the research priority exercise:

### 3.1 Step 1: Formation of the Steering Committee

A steering committee was established comprising academic epidemiologists and researchers in Reproductive, Maternal, Newborn, Child and Adolescent Health (RMNCAH), preconception health, perinatal and reproductive epidemiology, preventive care and health services. It also included clinicians such as obstetricians/gynaecologists and general practitioners with expertise in preconception and adolescent health in Australia. The committee also included a young interest‐holder, aged 18–24 years, who has lived in Australia for more than 6 months. The Committee was responsible for exploring the implications and scope of the PSP exercise, which involved reviewing the broader preconception health domains and the specific research questions that guided the evidence‐checking stage. The Committee was also responsible for advising on data management and quality assurance processes, overseeing interim prioritisation, approving the final shortlist of research questions, supporting dissemination of the final priorities and contributing to the development of research questions based on the agreed priorities and engagement with relevant interest‐holders.

### 3.2 Step 2: Conducting a Literature Search and Development of Questions

Research uncertainties were identified based on findings from a recent scoping review and evidence gap map that examined preconception health risk factors and interventions among adolescents and young adults to prevent adverse maternal, perinatal, and child health outcomes [5, 28]. To capture relevant evidence published since the completion of these reviews, an additional literature search was conducted on PubMed and Google Scholar using terms related to preconception health, adolescents, young adults, risk factors, interventions and outcomes. The search aimed to identify existing research gaps and priorities around the preconception health of adolescents and young adults (10–4 years) residing in Australia for more than 6 months. The literature published between 2010 and 2024 was reviewed, limited to English‐language publications only. Studies reporting on broader age groups and providing segregated data on adolescents and young adults were also included.

A comprehensive list of potential research priority questions was developed based on the literature search and subsequently categorised into domains around the WHO areas of preconception care and the Australian preconception health domains [1, 29], to facilitate review and prioritisation. The draft questionnaire was presented and shared with the steering committee on 17 March 2025, to ensure collective agreement on the proposed domains and questions. During this session, the committee reviewed and refined the proposed questions and domains based on their expertise, professional experience, and lived experiences. Additional questions were added to address important gaps identified by the committee, while questions that had been subsequently addressed in the literature or were considered no longer relevant were removed or modified. The domains were also further refined, named, and categorised to ensure they appropriately reflected the key areas of preconception health and the priorities of adolescents and young adults. Through structured discussions, 80 research questions were finalised under 10 domains (Box 1), thus capturing the nature and focus of each question.

Box 1 **Preconception Health Domains for Adolescents and Young Adults in Australia.**

**Domain 1:** Early Intervention and Prevention

**Domain 2:** Access to Preconception Care and the Healthcare System

**Domain 3:** Sexual and Reproductive Health (SRH) and Family Planning Services

**Domain 4:** Violence and Mental Health

**Domain 5:** Nutrition, Weight Management, and Physical Activity

**Domain 6:** Smoking, Tobacco, Alcohol, and Substance Use

**Domain 7:** Communicable and Non‐communicable Diseases (Including Infections)

**Domain 8:** Exposure to Environment and Chemicals

**Domain 9:** Genetics

**Domain 10:** Priority Populations (including First Nations, culturally and racially marginalised (CARM) and Lesbian, Gay, Bisexual, Transgender, Queer/Questioning, Intersex and Asexual/Aromantic plus (LGBTQIA+) populations and people with disability)

### 3.3 Step 3: Research Prioritisation‐ Survey 1/Phase I

The initial research priority exercise was conducted through an online survey conducted between 1 June 2025 and 20 September 2025. The survey aimed to gather responses from a representative and diverse group of professionals and young interest‐holders in Australia. The survey consisted of two parts. The first part included a participant information sheet, consent form, eligibility screening questions, sociodemographic data (such as age, gender and state), and a question on the appropriate timing for initiating preconception health promotion among adolescents and young adults. Occupation and years of experience were also asked of the profession interest‐holders. The second component of the survey focused on a research‐priority exercise comprising 80 questions across 10 domains (see Table S2). Participants were asked to rate each question on a 1–9 Likert scale, with *high (1–3)*, *medium (4–6)* or *low (7–9) priority*. This approach was adopted to offer clearer differentiation between importance levels, allowing participants to rate each question individually instead of ranking a large list of questions. Rating was deemed more suitable because ranking numerous questions might cause participants to improperly distinguish between questions that they find similar and important. Participants were also given the option to skip any question. The survey was fully anonymous, and no personal contact information was captured. Participants who took part in the second survey were not required to have completed the first survey.

As per the steering committee's suggestion, separate surveys were developed for professional and young interest‐holders using Qualtrics XM [30]. The survey of young interest‐holders contained the same questions but was written in plain language to ensure clarity and ease of understanding (See Table S3). The questionnaire was co‐designed with the Steering Committee and piloted with 10 participants who were not part of the Steering Committee. Graduate students at the Adelaide University conducted the pilot testing, including young adults. The pilot testing also ensured that the questionnaire for both interest‐holders had the same understanding and meaning of the questions. Following the pilot testing, questions were rephrased to improve readability and clarity, and additional definitions were included for medical terms as suggested to support understanding. The responses from the pilot test were excluded from the final analysis. The Steering Committee did not participate in conducting the pilot survey, and the study participants were not members of the Steering Committee.

#### 3.3.1 Participants and Recruitment

The participants in the online ranking of the questions included young interest‐holders, that is, adolescents and young adults (18–24 years) residing in Australia for more than 6 months. The targeted professional interest‐holders included healthcare professionals, academic researchers, teachers/lecturers, government officials and policymakers who had been working on preconception health or adolescent health in Australia for more than 6 months.

Professional and young interest‐holders were recruited through multiple channels. Study flyers and participant information sheets were disseminated via social media platforms such as Facebook, LinkedIn, Instagram, X and Bluesky to engage digitally connected professionals and young interest‐holders. In addition, materials were shared through WhatsApp groups of Muslim and Christian communities and health organisations, such as Healthy Development Adelaide, Preconception Health Network, SHINE SA, Youth Affairs Council Victoria, Survivors of Torture and Trauma Rehabilitation Services (STTARS) and the Consumer and Community Involvement Program (CCI), to facilitate access through trusted local networks. Students and the advisory group of adolescents and young people at the Adelaide University were also engaged. This multi‐channel approach aimed to build trust and ensure the inclusion of both professional and young interest‐holders.

To recruit professional interest‐holders, online searches were conducted on Google and Google Scholar to identify relevant organisations and individuals, published literature was reviewed to identify subject‐matter experts, and contacts established through public health conferences were drawn on. Direct email invitations were sent to identified individuals and organisations. Members of the steering committee further supported recruitment by sharing information within their professional networks. Some participants (professional interest‐holders) also connected us with additional experts in the field, helping us maximise our reach. Collectively, these strategies enabled engagement with professional interest‐holders across sectors involved in the care and health of adolescents and young adults.

### 3.4 Step 4: Data Management and Analysis—Refining Questions After Phase I of Research Prioritisation

After gathering responses from the first survey, all responses were exported from Qualtrics to an Excel sheet, which was stored on the Adelaide University's Box drive. The University's Box drive is encrypted and password‐protected, accessible only to those with whom the data is shared, and requires university login credentials to sign in.

Median scores were calculated for each rated question. This was done separately for responses received from professional interest‐holders and young interest‐holders. Median scores were also calculated for each question by combining responses from both professional and young interest‐holders. These scores highlighted uncertainties that were seen as priorities by different groups and where their views aligned or differed. After calculating median scores, all high‐prioritised questions (median scores of 1 to ≤ 3) were presented to the Steering Committee on 1 and 16 October 2025 (based on the availability of the Steering Committee members). Based on the first round of the survey, 24 questions (across five domains) were prioritised, reflecting questions rated highly by professional interest‐holders and young interest‐holders. The members highlighted a concern about excluding priority questions from the other five domains that were not highly prioritised. To address this concern, four more questions that were considered a priority in the combined score (i.e., Questions 6.2, 6.4, 7.3 and 7.5) from the unprioritised domains only were included in the list; as a result, 28 questions (across seven domains) were prioritised in the first round of the survey, which reflected the research priorities of both professional and young interest‐holders (See Table S2).

### 3.5 Step 5: Research Prioritisation—Survey 2/Phase II

Phase II was the final stage of the priority‐setting process to prioritise the top 10 research uncertainties identified through consensus on preconception health of adolescents and young adults in Australia.

Following Step 4, the top 28 research priorities were sent to the participants for re‐prioritisation. The process of recruitment of participants was similar to Phase I. It was not compulsory for participants to complete the Phase I survey to participate in Phase II. The second survey was conducted between 10 November 2025 and 30 January 2026. This survey also consisted of two parts. The first part included a participant information sheet, consent form, eligibility screening form, and sociodemographic questions. The second component of the survey focused on a research‐priority exercise, which consisted of prioritised questions across seven domains.

After gathering responses from participants in the Round 2 survey, questions were prioritised as described in Step 4. The top 10 priorities were shared with the steering committee members on 10 February 2026, and their insights and feedback helped prioritise the top 10 unanswered questions about the preconception health of adolescents and young adults residing in Australia.

## 4 Results

The initial survey captured 53 responses from professional interest‐holders and 44 from young interest‐holders. Seven participants from each group were ineligible to participate because they either had no experience or knowledge of the topic, did not understand English, or did not fall within the eligible age group of young interest‐holders, and 5 professional interest‐holders and 10 young interest‐holders either did not give consent or did not respond to the consent form. Nineteen responses from professional interest‐holders and 13 from young interest‐holders were incomplete, leaving 22 from professional interest‐holders and 14 from young interest‐holders in our final sample. In the second round of the survey, a total of 63 responses were captured from professional interest‐holders and 41 from young interest‐holders, of which 17 responses from professional interest‐holders and 7 from young interest‐holders were ineligible. Five young interest‐holders either did not give consent or did not respond to the consent form. Sixteen responses from professional interest‐holders and 15 from young interest‐holders were incomplete, leaving 30 complete responses from professional interest‐holders and 14 from young interest‐holders in our final sample for the second survey.

In the first round of the survey, most of the professional interest‐holders were female professionals from South Australia aged 25 years and above working in academia with 1–5 years of experience (Table 1). Most of the young interest‐holders in Round 1 were males aged 21–24 years, while Round 2 had an equal number of male and female respondents. Of the 22 professional interest‐holders, most suggested initiating preconception health promotion during early and late adolescence. On the contrary, most of the young interest‐holders reported starting preconception health promotion at young adulthood (20–24 years) or only when planning pregnancy.

**Table 1:** Characteristics of included participants.

Characteristics	Round 1		Round 2	
	Professional interest‐holders	Young interest‐holders	Professional interest‐holders	Young interest‐holders
	*N* = 22	*N* = 14	*N* = 30	*N* = 14
Age				
18–20 years	NA	2	NA	4
21–24 years	NA	12	NA	10
25–40 years	10	NA	10	NA
41–50 years	7	NA	11	NA
≥ 50 years	5	NA	8	NA
Prefer not to say	0	0	1	0
Gender				
Female	17	6	26	7
Male	5	8	4	7
Non‐binary/non‐conforming/transgender	0	0	0	0
States				
Australian Capital Territory	0	0	0	0
New South Wales	4	1	10	0
Northern Territory	0	0	0	0
Queensland	4	0	4	0
South Australia	8	12	10	12
Tasmania	0	0	0	0
Victoria	5	0	6	2
Western Australia	1	1	0	0
Occupation^a^				
Academic research position	15	NA	24	NA
Academic teaching position	1		3	
Healthcare professionals (doctor, nurse, counsellor, psychologist, etc.)	6		11	
Health system specialist	0		1	
Non‐government official	1		0	
Policymaker	3		1	
Program manager/Research manager	1		1	
Volunteer	0		1	
Experience				
≤ 1–5 years	12	NA	10	NA
6–10 years	3		2	
More than 10 years	7		18	
Time of starting preconception health promotion				
At the time of first sexual intercourse	3	0	NA	
During early adolescence (10–14 years)	11	1		
During late adolescence (15–19 years)	4	2		
At young adulthood (20–24 years)	1	7		
Only when planning pregnancy	0	4		
From early childhood (other)	1	0		
A life course approach (other)	1	0		
During adolescence (other)	1	0		

Abbreviation: NA, not applicable.

^a^ More than one occupation was selected by the participants.

### 4.1 Survey 1: Initial Prioritisation

Based on the first round of the survey, 28 questions (across seven domains) were prioritised, which are presented in Table 2. Most of the research questions were from the sexual and reproductive health (SRH) and family planning service domain (six questions), the violence and mental health domain (six questions), and the priority populations domain (six questions). This was followed by access to preconception care and the healthcare system (four questions), the early intervention and prevention domain (two questions), smoking, tobacco, alcohol and substance use (two questions), and the communicable and non‐communicable diseases domain (two questions). Genetics, exposure to the environment and/or chemicals, nutrition, weight management and physical activity domains were not highly prioritised by either professional or young interest‐holders.

**Table 2:** Domains and research questions prioritised in the first round of the survey.

Domains and questions			Median score		
			Combined	Professional interest‐holders	Young interest‐holders
Domain 1: Early intervention and prevention	1.3	What are the preconception risk factors among adolescents and young adults that can help prevent adverse maternal, perinatal and infant/child health outcomes?	2.0	2.0	3.0
	1.6	What are the effective strategies to engage and involve adolescents and young adults in improving their preconception health?	3.0	3.0	3.0
Domain 2: Access to preconception care and the healthcare system	2.2	How can we involve adolescents and young adults in planning, designing, and development of programmes to improve preconception healthcare?	3.0	3.0	2.5
	2.3	What strategies can be implemented to enhance the delivery, affordability, and coverage of preconception interventions across various settings, such as urban and rural areas?	3.0	3.0	3.0
	2.4	What are the most effective strategies for engaging adolescents and young adults in health education, including school‐based programmes and online platforms?	3.0	3.0	3.0
	2.5	What are the barriers preventing adolescents and young adults from accessing preconception care?	3.0	3.0	3.0
Domain 3: Sexual and reproductive health (SRH) and family planning services	3.1	What are the long‐term health impacts of coercion in reproductive decision‐making on perinatal, maternal and child health outcomes?	3.0	3.0	3.0
	3.2	To what extent do adolescents and young adults experience coercion in making decisions about pregnancy, such as being pressured to either continue with or terminate a pregnancy?	2.0	3.0	2.0
	3.3	What factors contribute to the pressure adolescents and young adults face when deciding whether to keep or end a pregnancy, and how can reproductive health policies and interventions help address these issues?	3.0	3.0	3.0
	3.5	What are the existing sexual and reproductive healthcare services/interventions and family planning services available for adolescents and young adults (all genders)?	3.0	3.0	3.0
	3.6	What is the knowledge among adolescents and young adults on existing family planning and sexual and reproductive health (SRH) services, and the accessibility to them?	3.0	3.0	2.5
	3.16	How effective are current school‐based SRH programmes in improving preconception health knowledge and behaviours?	2.5	3.0	2.0
Domain 4: Violence and mental health	4.1	What are the long‐term health impacts of violence (be it domestic, sexual or dating violence) during preconception on perinatal, maternal and child health outcomes?	3.0	3.0	3.0
	4.2	What is the impact of mental health issues at preconception on perinatal, maternal and child health outcomes?	2.0	3.0	2.0
	4.4	How can the detection and treatment of mental health disorders at preconception help improve pregnancy and child health outcomes?	3.0	3.0	2.5
	4.5	How can mental health screening and support be effectively integrated into routine preconception care for adolescents and young adults?	2.5	2.0	3.0
	4.7	What strategies could be developed that are effective in preventing intimate partner violence and coerced sex among adolescents and young adults?	2.0	2.0	2.5
	4.8	How do programmes designed to prevent and reduce gender‐based violence equip adolescents and young adults to address and reduce their risk?	3.0	3.0	3.0
Domain 6: Smoking, tobacco, alcohol and substance use	6.2	What are the long‐term impacts of preconception vaping on pregnancy and the health of future offspring?	3.0	3.0	3.5
	6.4	What are the long‐term impacts of recreational drug use during preconception on pregnancy and the health of future offspring?	3.0	3.0	3.5
Domain 7: Communicable and non‐communicable diseases (including infections)	7.3	What are the pre‐pregnancy predictors of pre‐eclampsia/eclampsia among young mothers?	3.0	3.5	3.0
	7.5	What are affordable, effective and feasible ways to screen adolescents and young adults for long‐term health issues, for example, high blood pressure, during the preconception period?	3.0	3.0	4.0
Domain 10: Priority populations	10.1	What are the specific preconception health needs and challenges faced by young migrants and refugees/culturally and racially marginalised (CARM)?	2.0	2.0	3.0
	10.2	What are the specific preconception health needs and challenges faced by young First Nations people?	3.0	2.5	3.0
	10.3	What are the specific preconception health needs and challenges faced by adolescents and young adults with disabilities?	2.0	2.0	3.0
	10.4	What are the specific preconception health needs and challenges faced by young LGBTQIA+ populations?	3.0	2.5	3.0
	10.6	How can preconception health services be better tailored to reach and engage First Nations adolescents and young adults?	2.0	2.0	2.5
	10.7	How can preconception health services be better tailored to reach and engage adolescents and young adults with a disability?	2.0	2.0	3.0

*Note:* Scoring: High priority: 1–3 score; Moderate priority: 4–6 score; Low priority: 7–9 score.

### 4.2 Survey 2: Prioritising the Top 10 Research Questions

After the second round of the survey, the top 10 questions from five domains were prioritised by both professional and young interest‐holders, which are presented in Table 3. Most of the research questions were from the violence and mental health domain (four questions), followed by the early intervention and prevention domain (two questions), smoking, tobacco, alcohol and substance use (two questions), access to preconception care and the healthcare system (one question) and the priority populations domain (one question). Communicable and non‐communicable diseases (including infections) and SRH and family planning service domains were prioritised by professional interest‐holders, but not by the young interest‐holders.

**Table 3:** Domains and research questions prioritised in the second round of the survey.

Domains and research questions			Median scores		
			Combined	Professional interest‐holders	Young interest‐holders
Domain 1: Early intervention and prevention	1.3	What are the preconception risk factors among adolescents and young adults that can help prevent adverse maternal, perinatal and infant/child health outcomes?	3	3	3
	1.6	What are the effective strategies to engage and involve adolescents and young adults in improving their preconception health?	2	2	3
Domain 2: Access to preconception care and the healthcare system	2.5	What are the barriers preventing adolescents and young adults from accessing preconception care?	2	2.5	2
Domain 4: Violence and mental health	4.2	What is the impact of mental health issues at preconception on perinatal, maternal and child health outcomes?	3	3	3
	4.4	How can the detection and treatment of mental health disorders at preconception help improve pregnancy and child health outcomes?	3	3	3
	4.5	How can mental health screening and support be effectively integrated into routine preconception care for adolescents and young adults?	3	2	3
	4.7	What strategies could be developed that are effective in preventing intimate partner violence and coerced sex among adolescents and young adults?	2	2	1
Domain 6: Smoking, tobacco, alcohol and substance use	6.2	What are the long‐term impacts of preconception vaping on pregnancy and the health of future offspring?	3	3	3
	6.4	What are the long‐term impacts of recreational drug use during preconception on pregnancy and the health of future offspring?	3	3	3
Domain 10: Priority populations	10.3	What are the specific preconception health needs and challenges faced by adolescents and young adults with disabilities?	2.5	2	3

*Note:* Scoring: High priority: 1–3 score; Moderate priority: 4–6 score; Low priority: 7–9 score.

Of the top 10 questions, the following two were highly prioritised by professionals and young interest holders, with median scores ranging from 1 to 2.5.

- *Domain 2: Access to Preconception Care and the Healthcare System*: What are the barriers preventing adolescents and young adults from accessing preconception care?
- *Domain 4: Violence and Mental Health:* What strategies could be developed that are effective in preventing intimate partner violence (IPV) and coerced sex among adolescents and young adults?

## 5 Discussion

The study identified the top 10 research priorities to improve the preconception health of adolescents and young adults in Australia. The highly ranked priorities centred on early intervention and prevention; access to preconception care and health systems; violence and mental health; smoking, tobacco, alcohol and substance use; and preconception health of priority populations, reflecting a shift from traditional biomedical framing toward broader social and behavioural determinants of health. Because these findings are based on a modest, self‐selected sample of interest‐holders, particularly a small and gender‐imbalanced group of young interest‐holders (*n* = 14) concentrated in South Australia, the interpretive comparisons drawn below between professional and young interest‐holder perspectives should be read as hypothesis‐generating rather than definitive, and as a starting point for confirmation in larger and more representative youth samples. This study also highlights a difference in opinions between the professional and young interest‐holders regarding the initiation of preconception care. Notably, professional interest‐holders recommended initiating preconception health promotion in early adolescence, whereas young interest‐holders preferred initiating it in young adulthood (20–24 years), suggesting differences in perceived relevance and readiness gap, as adolescents and young adults do not feel ready until their early 20s, while professionals suggest starting early, in middle school or high school. The lack of consensus can also be due to a lack of awareness or genuine differences in priorities perceived by young interest‐holders. A recent study from Australia reported a lack of awareness among the general population regarding preconception care [31]. Another study reported that only 53% of general practitioners were aware of the preconception care guidelines in Australia [32].

This study identified a set of shared research priorities but also reported on divergent perspectives among interest‐holder groups. Professional interest‐holders ranked communicable and non‐communicable diseases (including infections) and the SRH and family planning domains as high priorities, but these domains were not considered priorities by the young interest‐holders in the second round of the survey. This difference in perspective may reflect distinct ways in which professionals and young people perceive, experience, and understand the preconception health needs for adolescent and young adults. Nutrition, weight management and physical activity were not considered a high priority in the first round of the survey by both professionals and young interest‐holders. Despite their prominence in the preconception health frameworks [29, 33], these domains were not considered as a high priority by both groups, perhaps highlighting a mismatch with system‐level priorities. This misalignment has important implications for the design, acceptability, and uptake of preconception interventions. Genetics, environment and exposure to chemicals were also not prioritised in the first round of the survey; however, they pose a significant threat to maternal and child health. Current evidence also highlights the absence of evidence on environmental or climate exposure on child outcomes, as many studies on pre‐pregnancy cohorts have also failed to measure these variables as exposures [34].

Regarding the priority populations domain, questions on the specific preconception health needs and challenges among the adolescents and young adults with disability emerged in the final top 10 priorities, whereas First Nations, culturally and racially marginalised (CARM), and Lesbian, Gay, Bisexual, Transgender, Queer/Questioning, Intersex and Asexual/Aromantic plus (LGBTQIA+) populations did not, despite strong prioritisation by professional interest‐holders. The variation between professional interest‐holders' scores (consistently high) and young interest‐holders' scores (moderate) is interesting. The difference in how young interest‐holders ranked priority populations may reflect their perceived personal relevance rather than a lack of importance. As the demographic information on Indigenous status, ethnicity or disability was not collected, it is difficult to know whether priorities from these groups were diluted when responses were combined. Importantly, these groups should be viewed through a critical lens that must be integrated across all research activities, rather than as mutually exclusive or topic‑specific domains.

Apart from the differences in perspectives of the interest‐holder groups, it is important to note the close alignment of violence and mental health, and smoking, tobacco, alcohol and substance use domains. Several questions within these domains are interconnected, indicating a programmatic or implementation‐focused research approach rather than addressing each question in isolation. For example, Questions 4.2, 4.4, 4.5 and 4.7 from the violence and mental health domain represented a continuum, starting with exposure and impacts to identification, integration into care and prevention. Similarly, Questions 6.2 and 6.4 on the long‐term effects of preconception exposure to vaping and recreational drug use from the smoking, tobacco, alcohol, and substance use domain suggest opportunities to be studied together using similar study designs or cohorts.

Finally, the research priorities centred on violence and mental health; early preconception health intervention and prevention; substance use, access to care and priority populations. It is not surprising that violence was a high priority area for both interest groups, given the rates of sexual and physical assault experienced by young women [35]. Exposure to violence or abuse during childhood or before pregnancy has been reported as a strong predictor of family and domestic violence during pregnancy, with poor consequences on maternal and perinatal health outcomes [36]. According to the current evidence, young Australian women are more at risk of sexual assault compared to older women, resulting in an unwanted pregnancy or termination [36, 37]. A study on pregnant Indigenous women or those with a refugee status in Brisbane, Australia, reported higher rates of IPV compared to non‐Indigenous women and those with no refugee status [38]. Also, those exposed to IPV reported higher odds of pregnancy complications, small for gestational age (SGA) babies, premature birth, and neonatal mortality [38]. According to existing evidence, COVID‐19, social media, financial uncertainty and political instability have raised levels of distress among young Australians [39]. This emphasises why both groups prioritised questions related to violence, highlighting the importance of preconception interventions to address violence, especially among adolescents and young adults.

The other identified priorities also align well with the emerging evidence highlighting mental health burden and substance use as key concerns amongst young Australians [40, 41]. The Australian Institute of Health and Welfare reported substance use disorders, mental health and injuries as major contributors to the high burden of disease among young Australians aged 15–24 years in 2023 [42]. Similarly, a recent study using the Global Burden of Disease Study 2021 data also reported alcohol and drug use as the leading risk factors for death among adolescents and young adults (10–24 years) in Australia [41]. Beyond disease burden, these priorities reflect lived experiences shaped by social, economic and structural factors, including cost‐of‐living pressures, peer influences and barriers to accessing care [40], underscoring the need for evidence strengthening around preconception health of adolescents and young adults, and emphasising the direction of resources to not only improve their health but also the health of families.

The strength of this study is that it captures young interest‐holders' responses, and, to our knowledge, it is the first research priority exercise identifying priority research areas for improving preconception health among adolescents and young adults in Australia. The study has several limitations. First, most responses from the study participants came from South Australia, with very few from other states of Australia, therefore limiting the generalisability of the study findings. Second, the survey did not collect data on ethnicity, education, First Nations or disability status. Third, due to time constraints, youth advisory group members from the Adelaide University were invited to join the Steering Committee, but only one member agreed to join the Steering Committee, leading to limited representation of young adults and the opportunity to contribute. Fourth, the survey involved a relatively small number of young interest‐holders, with imbalanced gender representation among them. Therefore, the perspectives gathered may not fully represent the diversity of adolescents and young adults in Australia. Hence, the results should be interpreted with caution when drawing conclusions about youth priorities. Fifth, the study approached youth organisations to capture the views of adolescents and young adults in the priority‐setting process; however, these organisations may represent particular interests or experiences related to youth health and reproductive issues. As a result, the perspectives obtained may not fully reflect the diversity of all adolescents and young adults in Australia, especially those not involved with youth organisations or health programmes. Sixth, several research questions considered a priority by professional interest‐holders were not considered a priority by young interest‐holders, which may be due to a lack of knowledge and differences in socio‐economic and cultural backgrounds. There is also a possibility that some of the groups, such as First Nations, LGBTQIA+ and CARM communities, may be underrepresented. Finally, this study used a modified JLA approach. In Phase 1, participants were invited to prioritise the research questions listed in the survey rather than propose new questions. This modification deviates from the standard JLA method, which may have limited the opportunity for the interest‐holders to contribute additional ideas or suggest new research questions.

## 6 Conclusion

The study identified 10 research priorities to improve preconception health of adolescents and young adults in Australia, which centred on violence and mental health; early preconception health intervention and prevention; substance use, access to care, and priority populations. This exercise encourages new research and collaboration, and provides a guide for future research investments, policy development and design of preconception interventions across different settings. Translating these priorities into research has the potential not only to improve the health outcomes of adolescents but also of their future generations.

## Author Contributions

**Zahra Ali Padhani:** conceptualisation, methodology, data curation, formal analysis, investigation, visualisation, project administration, resources, writing – original draft, writing – review and editing, funding acquisition. **Jodie C. Avery:** conceptualisation, methodology, validation, supervision, writing – review and editing, investigation. **Gizachew A. Tessema:** conceptualisation, methodology, investigation, writing – review and editing, validation, supervision. **Keeth L. B. Mayakaduwage:** methodology, investigation, validation, formal analysis, project administration, visualisation, writing – original draft, writing – review and editing, data curation, supervision. **Jacqueline A. Boyle:** writing – original draft, writing – review and editing, formal analysis, investigation, validation. **Danielle Mazza:** writing – review and editing, writing – original draft, formal analysis, investigation, validation. **Sara Ataie:** writing – original draft, writing – review and editing, formal analysis, investigation, validation. **Salima Meherali:** writing – review and editing, conceptualisation, methodology, supervision. **Zohra S. Lassi:** conceptualisation, writing – review and editing, writing – original draft, visualisation, validation, methodology, supervision, investigation, formal analysis.

## Ethics Statement

Ethical approval was obtained from the Human Research Ethics Committee at the University of Adelaide, now known as Adelaide University (H‐2024‐176) on 24 September 2024. After the first steering committee meeting, the survey was updated, and an ethics amendment was requested, which was approved on 2 May 2025.

## Conflicts of Interest

The authors declare no conflicts of interest.

## Supporting information


Supplementary File

## Data Availability

All data associated with the study are provided in the manuscript and the Supporting Information.
