# Supplementary material for: Preconception Health Research Priorities for Adolescents and Young Adults in Australia
Source: Health Expect. 2026 Oct 28;29(6):e70950. doi: 10.1111/hex.70950 (PMC13714562; doi:10.1111/hex.70950)
Supplement: Supplementary file 1 [file HEX-29-e70950-s001.docx]

**Supplementary File**

**S-Table 1: Guidance for Reporting Involvement of Patients and the Public (GRIPP 2) checklist**

| Section and topic | Item | Reported on page No |
| --- | --- | --- |
| Section 1: Abstract of paper | | |
| 1a: Aim | Report the aim of the study | 2 |
| 1b: Methods | Describe the methods used by which patients and the public were involved |  |
| 1c: Results | Report the impacts and outcomes of PPI in the study |  |
| 1d:Conclusions | Summarise the main conclusions of the study |  |
| 1e: Keywords | Include PPI, “patient and public involvement,” or alternative terms as keywords |  |
| Section 2: Background to paper | | |
| 2a: Definition | Report the definition of PPI used in the study and how it links to comparable studies |  |
| 2b: Theoretical underpinnings | Report the theoretical rationale and any theoretical influences relating to PPI in the study | Not applicable |
| 2c: Concepts and theory development | Report any conceptual models or influences used in the study | Not applicable |
| Section 3: Aims of paper | | |
| 3: Aim | Report the aim of the study | 4 |
| Section 4: Methods of paper | | |
| 4a: Design | Provide a clear description of methods by which patients and the public were involved | Research priority-setting exercise was conducted following the James Lind Alliance (JLA) methodology and details are provided on page 4 |
| 4b: People involved | Provide a description of patients, carers, and the public involved with the PPI activity in the study | 6 |
| 4c: Stages of involvement | Report on how PPI is used at different stages of the study | 4-7 |
| 4d: Level or nature of involvement | Report the level or nature of PPI used at various stages of the study |  |
| Section 5: Capture or measurement of PPI impact | | |
| 5a: Qualitative evidence of impact | If applicable, report the methods used to qualitatively explore the impact of PPI in the study | Not applicable |
| 5b: Quantitative evidence of impact | If applicable, report the methods used to quantitatively measure or assess the impact of PPI | 6-7 |
| 5c: Robustness of measure | If applicable, report the rigour of the method used to capture or measure the impact of PPI | Not applicable |
| Section 6: Economic assessment | | |
| 6: Economic assessment | If applicable, report the method used for an economic assessment of PPI | Not applicable |
| Section 7: Study results | | |
| 7a: Outcomes of PPI | Report the results of PPI in the study, including both positive and negative outcomes | Results are reported according to JLA methodology from pages 7-8 |
| 7b: Impacts of PPI | Report the positive and negative impacts that PPI has had on the research, the individuals involved (including patients and researchers), and wider impacts |  |
| 7c: Context of PPI | Report the influence of any contextual factors that enabled or hindered the process or impact of PPI |  |
| 7d: Process of PPI | Report the influence of any process factors, that enabled or hindered the impact of PPI |  |
| 7ei: Theory development | Report any conceptual or theoretical development in PPI that have emerged | Not Applicable |
| 7eii: Theory development | Report testing of theoretical models, if any | Not Applicable |
| 7f: Measurement | If applicable, report all aspects of instrument development and testing (eg, validity, reliability, feasibility, acceptability, responsiveness, interpretability, appropriateness, precision) | Not Applicable |
| 7g: Economic assessment | Report any information on the costs or benefit of PPI | Not Applicable |
| Section 8: Discussion and conclusions | | |
| 8a: Outcomes | Comment on how PPI influenced the study overall. Describe positive and negative effects | 8 |
| 8b: Impacts | Comment on the different impacts of PPI identified in this study and how they contribute to new knowledge | 9-10 |
| 8d: Theoretical underpinnings | Comment on any way your study adds to the theoretical development of PPI | Not Applicable |
| 8e: Context | Comment on how context factors influenced PPI in the study | 8-10 |
| 8f: Process | Comment on how process factors influenced PPI in the study |  |
| 8g: Measurement and capture of PPI impact | If applicable, comment on how well PPI impact was evaluated or measured in the study | Not Applicable |
| 8h: Economic assessment | If applicable, discuss any aspects of the economic cost or benefit of PPI, particularly any suggestions for future economic modelling. | Not Applicable |
| 8i: Reflections/critical perspective | Comment critically on the study, reflecting on the things that went well and those that did not, so that others can learn from this study | 10 |
| PPI=patient and public involvement | | |

**S-Table 2: Domains and research questions prioritised in the first round of the survey**

| **Domains and questions** | | | **Combined** | **Professional Interest-holders** | **Young Interest-holders** |
| --- | --- | --- | --- | --- | --- |
|  |  |  | **Median** | **Median** | **Median** |
| Domain 1: Early Intervention and Prevention | 1.1 | How do preconception health status and behaviours influence intergenerational health outcomes? | 3.0 | 3.0 | 3.5 |
|  | 1.2 | What is the understanding of preconception health among adolescents and young adults? | 3.0 | 3.0 | 4.0 |
|  | 1.3 | What are the preconception risk factors among adolescents and young adults that can help prevent adverse maternal, perinatal, and infant/child health outcomes? | 2.0 | 2.0 | 3.0 |
|  | 1.4 | When is the best time to offer preconception interventions to support better maternal, perinatal, and infant/child health outcomes? | 3.0 | 3.0 | 3.5 |
|  | 1.5 | What should be included in a core set of preconception health interventions for adolescents and young adults? | 3.0 | 3.0 | 4.5 |
|  | 1.6 | What are the effective strategies to engage and involve adolescents and young adults in improving their preconception health? | 3.0 | 3.0 | 3.0 |
|  | 1.7 | What are the social and economic implications of improving preconception health among adolescents and young adults? | 4.0 | 3.5 | 4.0 |
|  | 1.8 | What indicators should be used to monitor and evaluate preconception health status among adolescents and young adults? | 4.0 | 4.0 | 4.0 |
|  | 1.9 | How data collection methods should be used, and how can they be improved to better track preconception health outcomes among adolescents and young adults? | 4.0 | 4.0 | 5.0 |
| Domain 2: Access to Preconception Care and the Healthcare System | 2.1 | How can adolescent health services be better integrated with maternal care to ensure continuity of care for adolescents who become pregnant and transition into maternal health services? | 3.0 | 3.0 | 3.5 |
|  | 2.2 | How can we involve adolescents and young adults in planning, designing, and development of programs to improve preconception health care? | 3.0 | 3.0 | 2.5 |
|  | 2.3 | What strategies can be implemented to enhance the delivery, affordability, and coverage of preconception interventions across various settings, such as urban and rural areas? | 3.0 | 3.0 | 3.0 |
|  | 2.4 | What are the most effective strategies for engaging adolescents and young adults in health education, including school-based programs and online platforms? | 3.0 | 3.0 | 3.0 |
|  | 2.5 | What are the barriers preventing adolescents and young adults from accessing preconception care? | 3.0 | 3.0 | 3.0 |
|  | 2.6 | What role can digital health play in improving access to preconception care? | 4.0 | 3.5 | 4.5 |
|  | 2.7 | What are the feasibility and costs of implementing digital and other health information technologies to enhance preconception health? What are the key barriers to implementing these technologies? | 4.5 | 4.0 | 5.0 |
|  | 2.8 | What are the harmful impacts of digital platforms on adolescents and young adults, and how can these platforms be used to prevent negative behaviours or health outcomes? | 4.0 | 4.0 | 4.5 |
|  | 2.9 | What role do Participatory Learning and Action (PLA) interventions play in improving access to health services for adolescents and young adults? (Note: PLA is a participatory methodology where community members are actively involved in analysing their own situations, learning together, and taking action to address problems [Ref: Gosling and Edwards 2003]) | 4.0 | 4.0 | 4.0 |
|  | 2.10 | How effective are Participatory Learning and Action (PLA) interventions in improving adolescent and young adult access to healthcare, and what are the key barriers to their implementation? (Note: PLA is a participatory methodology where community members are actively involved in analysing their own situations, learning together, and taking action to address problems [Ref: Gosling and Edwards 2003]) | 4.0 | 4.5 | 3.0 |
| Domain 3: Sexual Reproductive Health and Family Planning Service | 3.1 | What are the long-term health impacts of coercion in reproductive decision-making on perinatal, maternal and child health outcomes? | 3.0 | 3.0 | 3.0 |
|  | 3.2 | To what extent do adolescents and young adults experience coercion in making decisions about pregnancy, such as being pressured to either continue with or terminate a pregnancy? | 2.0 | 3.0 | 2.0 |
|  | 3.3 | What factors contribute to the pressure adolescents and young adults face when deciding whether to keep or end a pregnancy, and how can reproductive health policies and interventions help address these issues? | 3.0 | 3.0 | 3.0 |
|  | 3.4 | What are the practices of female genital mutilation, and how do they impact maternal, perinatal, and child health outcomes | 4.0 | 4.0 | 4.0 |
|  | 3.5 | What are the existing sexual and reproductive healthcare services/interventions and family planning services available for adolescents and young adults (all genders)? | 3.0 | 3.0 | 3.0 |
|  | 3.6 | What is the knowledge among adolescents and young adults on existing family planning and sexual reproductive health (SRH) services and the accessibility to it? | 3.0 | 3.0 | 2.5 |
|  | 3.7 | How do migration status, cultural identity and norms impact the SRH experiences and decision-making behaviours of adolescents and young adults (of all genders)? | 3.0 | 2.5 | 3.5 |
|  | 3.8 | What are the family planning methods utilised by adolescents and young adults (all genders) to prevent unintended pregnancies? | 3.5 | 3.5 | 3.5 |
|  | 3.9 | What are the family planning methods utilised by adolescents and young adults (all genders) to prevent sexually transmitted diseases/infections? | 4.0 | 4.0 | 3.5 |
|  | 3.10 | What are the experiences and preferences of adolescents and young adults regarding contraceptive methods and decision-making? | 4.0 | 3.5 | 5.5 |
|  | 3.11 | What are the perceptions and attitudes of parents/guardians towards family planning and contraception for their children? | 4.0 | 4.0 | 4.0 |
|  | 3.12 | What approaches can be developed and adopted to increase awareness and improve access to emergency contraception? | 3.5 | 3.5 | 3.5 |
|  | 3.13 | Which community-based programs (for example: youth mentoring programs) can help improve SRH outcomes among females? | 3.5 | 3.0 | 4.5 |
|  | 3.14 | Which community-based programs (for example: youth mentoring programs) can help improve SRH outcomes among males? | 3.0 | 3.0 | 4.5 |
|  | 3.15 | Which community-based programs (for example: youth mentoring programs) can help improve SRH outcomes among the LGBTQIA+ population? | 3.0 | 3.0 | 3.5 |
|  | 3.16 | How effective are current school-based sexual and reproductive health programs in improving preconception health knowledge and behaviours? | 2.5 | 3.0 | 2.0 |
| Domain 4: Violence and Mental Health | 4.1 | What are the long-term health impacts of violence (be it domestic, sexual, or dating violence) during preconception on perinatal, maternal and child health outcomes? | 3.0 | 3.0 | 3.0 |
|  | 4.2 | What is the impact of mental health issues at preconception on perinatal, maternal and child health outcomes? | 2.0 | 3.0 | 2.0 |
|  | 4.3 | How does the use of anti-depressants and anti-anxiety medications during the preconception period impact maternal, perinatal, and child health outcomes? | 3.0 | 4.0 | 3.0 |
|  | 4.4 | How can the detection and treatment of mental health disorders at preconception help improve pregnancy and child health outcomes? | 3.0 | 3.0 | 2.5 |
|  | 4.5 | How can mental health screening and support be effectively integrated into routine preconception care for adolescents and young adults? | 2.5 | 2.0 | 3.0 |
|  | 4.6 | How can community health workers effectively and feasibly deliver a preventive mental health intervention package, with linkages to the primary health care system for treatment? | 3.0 | 3.0 | 4.0 |
|  | 4.7 | What strategies could be developed that are effective in preventing intimate partner violence and coerced sex among adolescents and young adults? | 2.0 | 2.0 | 2.5 |
|  | 4.8 | How do programs designed to prevent and reduce gender-based violence equip adolescents and young adults to address and reduce their risk? | 3.0 | 3.0 | 3.0 |
| Domain 5: Nutrition, Weight Management, and Physical Activity | 5.1 | What is the impact of preconception weight (underweight, overweight or obesity) and lifestyle during adolescence on maternal and perinatal health outcomes? | 4.0 | 4.0 | 4.5 |
|  | 5.2 | What is the impact of preconception weight (underweight, overweight or obesity) and lifestyle during adolescence on the growth and development of their children? | 5.0 | 5.0 | 6.0 |
|  | 5.3 | What is the impact of preconception dietary patterns and physical activity on pregnancy and birth outcomes? | 4.0 | 5.0 | 3.5 |
|  | 5.4 | What is the impact of preconception dietary quality during adolescence on pregnancy and birth outcomes? | 4.0 | 4.0 | 3.5 |
|  | 5.5 | What are the existing nutrition interventions targeted at adolescents and young adults during preconception to improve their maternal, perinatal, and child health outcomes? | 4.0 | 4.0 | 5.0 |
| Domain 6: Smoking, Tobacco, Alcohol, and Substance Use | 6.1 | What are the long-term effects of preconception exposure to tobacco smoke on pregnancy and the health of future offspring? | 4.0 | 5.0 | 3.0 |
|  | 6.2 | What are the long-term impacts of preconception vaping on pregnancy and the health of future offspring? | 3.0 | 3.0 | 3.5 |
|  | 6.3 | What are the long-term impacts of preconception alcohol use on pregnancy and the health of future offspring? | 4.0 | 5.0 | 4.0 |
|  | 6.4 | What are the long-term impacts of recreational drug use during preconception on pregnancy and the health of future offspring? | 3.0 | 3.0 | 3.5 |
|  | 6.5 | What is the impact of preconception interventions to prevent smoking among adolescents and young adults on pregnancy outcomes? | 4.0 | 4.0 | 4.0 |
|  | 6.6 | What is the impact of preconception interventions to prevent vaping among adolescents and young adults on pregnancy outcomes? | 4.0 | 2.0 | 5.0 |
|  | 6.7 | What is the impact of preconception interventions to prevent alcohol use among adolescents and young adults and its impact on pregnancy outcomes? | 4.0 | 4.0 | 3.5 |
|  | 6.8 | What is the impact of preconception interventions to prevent recreational drug use among adolescents and young adults and its impact on pregnancy outcomes? | 4.0 | 3.0 | 4.0 |
| Domain 7: Communicable and Non-communicable Diseases (Including Infections) | 7.1 | What are the pre-pregnancy predictors of gestational diabetes among young mothers? | 4.0 | 4.0 | 3.0 |
|  | 7.2 | What are the pre-pregnancy predictors of gestational hypertension among young mothers? | 4.0 | 4.0 | 4.0 |
|  | 7.3 | What are the pre-pregnancy predictors of pre-eclampsia/eclampsia among young mothers? | 3.0 | 3.5 | 3.0 |
|  | 7.4 | How does the use of medications for conditions like epilepsy or cardiac problems during the preconception period affect maternal, perinatal, and child health outcomes? | 4.0 | 4.0 | 4.0 |
|  | 7.5 | What are affordable, effective, and feasible ways to screen adolescents and young adults for long-term health issues, for example, high blood pressure, during the preconception period? | 3.0 | 3.0 | 4.0 |
|  | 7.6 | What are affordable, effective, and feasible ways to screen adolescents and young adults for infections during the preconception period? | 4.0 | 4.0 | 4.5 |
|  | 7.7 | What strategies are available to improve STI/HIV prevention and management as part of preconception care? | 4.0 | 4.0 | 4.0 |
|  | 7.8 | How does HPV vaccination during adolescence affect maternal, perinatal, and childhood health outcomes? | 4.5 | 4.0 | 5.5 |
| Domain 8: Exposure to Environment and Chemicals | 8.1 | What is the impact of environmental and chemical exposures during preconception on maternal, perinatal and child health? | 4.0 | 4.5 | 4.0 |
|  | 8.2 | What is the impact of preconception occupational exposures to cleaning products and disinfectants on maternal, perinatal and child health outcomes? | 4.0 | 4.0 | 4.0 |
|  | 8.3 | What public health policies and strategies are in place to regulate and reduce environmental exposures and harmful chemicals? | 4.0 | 5.0 | 4.0 |
|  | 8.4 | What strategies or approaches can help modify individuals' behaviour to reduce environmental exposure to prevent long-term health impacts? | 4.0 | 4.5 | 4.0 |
| Domain 9: Genetics | 9.1 | What are the preconception genetic risk factors among adolescents and young adults that can affect pregnancy and child health outcomes? | 5.0 | 5.0 | 4.5 |
|  | 9.2 | What is the role of genetic testing during preconception and its impact on pregnancy and child health outcomes? | 5.0 | 5.0 | 4.5 |
|  | 9.3 | How aware are adolescents and young adults of genetic screening options and genetic counselling, and how accessible are these services to them, especially in relation to preconception health? | 4.0 | 4.0 | 4.5 |
|  | 9.4 | What is the feasibility and cost-effectiveness of implementing community-based genetic screening programs? | 5.0 | 5.0 | 4.5 |
| Domain 10: Priority Populations | 10.1 | What are the specific preconception health needs and challenges faced by young migrants and refugees/culturally and racially marginalised (CARM)? | 2.0 | 2.0 | 3.0 |
|  | 10.2 | What are the specific preconception health needs and challenges faced by young First Nations people? | 3.0 | 2.5 | 3.0 |
|  | 10.3 | What are the specific preconception health needs and challenges faced by adolescents and young adults with disabilities? | 2.0 | 2.0 | 3.0 |
|  | 10.4 | What are the specific preconception health needs and challenges faced by young LGBTQIA+ populations? | 3.0 | 2.5 | 3.0 |
|  | 10.5 | How can preconception health services be better tailored to reach and engage adolescents and young adults from CARM, and migrant and refugee backgrounds? | 2.0 | 2.0 | 4.0 |
|  | 10.6 | How can preconception health services be better tailored to reach and engage First Nations adolescents and young adults? | 2.0 | 2.0 | 2.5 |
|  | 10.7 | How can preconception health services be better tailored to reach and engage adolescents and young adults with a disability? | 2.0 | 2.0 | 3.0 |
|  | 10.8 | How can preconception health services be better tailored to reach and engage young LGBTQIA+ populations? | 2.0 | 2.0 | 3.5 |
| Scoring:   - High priority: 1–3 score - Moderate priority:4–6 score - Low priority: 7–9 score | | | | | |

**S-Table 2: Domains and research questions in plain language for adolescents and young adults**

**Instructions:**

**All research questions refer to the preconception health of adolescents and young adults aged 10-24 years residing in Australia.**

Please rate each question's priority using the following scale:
High Priority (1-3): Urgent implementation needed; critical for immediate healthcare improvement
Moderate Priority (4-6): Important but less time-sensitive; should be addressed in the medium term
Low Priority (7-9): Beneficial but not urgent; can be addressed after higher priorities

The questionnaire is organised into different domains, with multiple questions in each section. For each question, please assign a numerical rating from 1 to 9 based on the scale above. You may prefer not to answer any question or exit the survey at any point.

Some important definitions before we begin:

**Preconception health:** defined as the health and nutrition of both men and women before getting pregnant

**Preconception period/preconception:** time before pregnancy

**Intervention:** defined as a treatment, procedure, or other action taken to prevent or treat disease, or improve health in other ways.

**Adolescence:** It is the phase of life between childhood and adulthood, from ages 10 to 19

**Young adults:** Individuals aged 18 to 25 years of age

**Perinatal period**: The perinatal period starts at 20 weeks of pregnancy and ends 28 days after the baby is born.

**Domain 1: Early Intervention and Prevention**

Based on your knowledge and experience, rank how important each question is for further research to improve the preconception health of adolescents and young adults (10-24 years) residing in Australia.

Please rate each question's priority using the following scale:
High Priority (1-3): Urgent implementation needed; critical for immediate healthcare improvement
Moderate Priority (4-6): Important but less time-sensitive; should be addressed in the medium term
Low Priority (7-9): Beneficial but not urgent; can be addressed after higher priorities

Definitions
**Preconception health:** defined as the health and nutrition of both men and women before getting pregnant
**Preconception period/preconception:** time before pregnancy
**Risk factors:** Health risk factors are attributes, characteristics or exposures that increase the likelihood of a person developing a disease or health disorder [Ref: AIHW]
**Intervention:** It is defined as a treatment, procedure, or other action taken to prevent or treat disease, or improve health in other ways.

| **Domains and questions** | | |
| --- | --- | --- |
|  |  |  |
| Domain 1: Early Intervention and Prevention | 1.1 | How do the health and health behaviours of adolescents and young adults before pregnancy influence the health of future generations? |
|  | 1.2 | What is the understanding of preconception health among adolescents and young adults? |
|  | 1.3 | What are the risk factors before pregnancy among adolescents and young adults that could help reduce the health problems of mothers during pregnancy, and improve the health of their babies and children? |
|  | 1.4 | When is the best time to provide preconception care (care before pregnancy) or support to improve the health of mothers, babies, and children? |
|  | 1.5 | What should be included in a basic package of preconception care for adolescents and young adults? |
|  | 1.6 | What are the most effective strategies to engage and involve adolescents and young adults in improving their preconception health? |
|  | 1.7 | How could improving the health of adolescents and young adults before pregnancy affect society and the economy? |
|  | 1.8 | What indicators/health factors should be used to monitor and evaluate preconception health status among adolescents and young adults? |
|  | 1.9 | How data collection methods should be used, and how can it be improved to better track preconception health outcomes among adolescents and young adults? |

**Domain 2: Access to Preconception Care and the Healthcare System**

Based on your knowledge and experience, rank how important each question is for further research to improve access to preconception care for adolescents and young adults (10-24 years) residing in Australia.

Please rate each question's priority using the following scale:
High Priority (1-3): Urgent implementation needed; critical for immediate healthcare improvement
Moderate Priority (4-6): Important but less time-sensitive; should be addressed in the medium term
Low Priority (7-9): Beneficial but not urgent; can be addressed after higher priorities

Definitions
**Participatory Learning and Action (PLA)** is a participatory methodology where community members are actively involved in analysing their own situations, learning together, and taking action to address problems [Ref: Gosling and Edwards 2003]

| Domain 2: Access to Preconception Care and the Healthcare System | 2.1 | How can health services for adolescents be better connected with maternal care to ensure smooth support for adolescents and young adults who become pregnant and transition into pregnancy and motherhood care? |
| --- | --- | --- |
|  | 2.2 | How can we involve adolescents and young adults in planning, designing, and development of programs to improve preconception health care? |
|  | 2.3 | What approaches or strategies can be taken or developed to improve delivery, increase coverage and affordability of preconception interventions in different settings (i.e., remote or non-remote areas)? |
|  | 2.4 | What are the most effective strategies for engaging adolescents and young adults in health education, including school-based programs and online platforms? |
|  | 2.5 | What are the barriers/challenges faced by adolescents and young adults while accessing preconception care? |
|  | 2.6 | What role can digital health play in improving access to preconception care? |
|  | 2.7 | What is the cost and feasibility of using digital technologies and other information technologies to improve the preconception health? What are the key barriers to implementing these technologies? |
|  | 2.8 | What are the harmful impacts of digital platforms on adolescents and young adults, and how can these platforms be used to prevent negative behaviours or health outcomes? |
|  | 2.9 | What role do Participatory Learning and Action (PLA) interventions play in improving access to health services for adolescents and young adults? (Note: PLA is a participatory methodology where community members are actively involved in analysing their own situations, learning together, and taking action to address problems [Ref: Gosling and Edwards 2003]) |
|  | 2.10 | How effective are Participatory Learning and Action (PLA) interventions in improving adolescent and young adult access to healthcare, and what are the key barriers/challenges to their implementation? (Note: PLA is a participatory methodology where community members are actively involved in analysing their own situations, learning together, and taking action to address problems [Ref: Gosling and Edwards 2003]) |

**Domain 3: Sexual Reproductive Health and Family Planning Service**

Based on your knowledge and experience, rank how important each question is for further research on improving the sexual and reproductive health of adolescents and young adults (10-24 years) residing in Australia.

Please rate each question's priority using the following scale:
High Priority (1-3): Urgent implementation needed; critical for immediate healthcare improvement
Moderate Priority (4-6): Important but less time-sensitive; should be addressed in the medium term
Low Priority (7-9): Beneficial but not urgent; can be addressed after higher priorities

**Trigger Warning!**
Some questions or topics in this domain relate to sensitive issues such as coercion, termination, and female genital mutilation (FGM). These may be distressing for individuals with past experiences of trauma. If you find any section uncomfortable, you are welcome to skip that part or choose "Prefer not to answer." Your well-being comes first.

Definitions
**Sexual health:** defined as “…a state of physical, emotional, mental and social well-being in relation to sexuality; it is not merely the absence of disease, dysfunction or infirmity. Sexual health requires a positive and respectful approach to sexuality and sexual relationships, as well as the possibility of having pleasurable and safe sexual experiences, free of coercion, discrimination and violence. For sexual health to be attained and maintained, the sexual rights of all persons must be respected, protected and fulfilled.” (Source: WHO, 2006a)

**Reproductive health includes:**
- a state of physical, mental and social wellbeing in matters relating to the reproductive system
- the ability to have a safe and satisfying sex life
- the ability and freedom to have a child if, when and how often you choose to
- the ability to have a healthy pregnancy, safe delivery and healthy baby. (Source: Commonwealth of Australia | Department of Health and Aged Care)

**Family planning** allows people to attain their desired number of children, if any, and to determine the spacing of their pregnancies. It is achieved through use of contraceptive methods and the treatment of infertility. (Source: WHO 2024)

**Female Genital Cutting/Mutilation:** refers to the practice of cutting the female private parts for non-medical reasons. It is a harmful tradition that can cause serious health problems, including pain, infections, and complications during childbirth.

**LGBTQIA+:** The **LGBTQIA+** acronym stands for Lesbian, Gay, Bisexual, Transgender, Queer, Intersex, Asexual, and the "+" represents other identities not explicitly mentioned within the acronym, encompassing a broad spectrum of sexual orientations, gender identities, and sex characteristics.

| Domain 3: Sexual Reproductive Health and Family Planning Service | 3.1 | What are the long-term health impacts of coercion/enforcement/pressure in reproductive decision-making on the health of mothers and their children? |
| --- | --- | --- |
|  | 3.2 | To what extent do adolescents and young adults experience coercion/enforcement/pressure in making decisions about pregnancy, such as being pressured to either continue with or terminate a pregnancy? |
|  | 3.3 | What factors contribute to the pressure adolescents and young adults face when deciding whether to keep or end a pregnancy, and how can reproductive health policies and interventions help address these issues? |
|  | 3.4 | What are the practices of female genital mutilation, and how do they impact the health of mothers and their children? |
|  | 3.5 | What are the existing sexual and reproductive healthcare services/interventions and family planning services available for adolescents and young adults (all genders)? |
|  | 3.6 | What is the knowledge among adolescents and young adults on existing sexual reproductive health and family planning services, and the accessibility to it? |
|  | 3.7 | How do migration status, cultural identity and norms impact the sexual reproductive health experiences and decision-making behaviours of adolescents and young adults (of all genders)? |
|  | 3.8 | What are the family planning methods utilised by adolescents and young adults (all genders) to prevent unintended/unwanted pregnancies? |
|  | 3.9 | What are the family planning methods utilised by adolescents and young adults (all genders) to prevent sexually transmitted diseases/infections? |
|  | 3.10 | What are the experiences and preferences of adolescents and young adults regarding birth control methods and decision-making? |
|  | 3.11 | What are the perceptions and attitudes of parents/guardians towards family planning and birth control for their children? |
|  | 3.12 | What strategies could be developed to promote knowledge of and access to emergency contraception/birth control? (Note: Emergency contraception, sometimes called the "morning after pill," is used to prevent pregnancy after unprotected sex or if contraception fails.) |
|  | 3.13 | Which community-based programs (such as youth mentoring programs and after-school programs) effectively promote positive sexual and reproductive health outcomes among females? |
|  | 3.14 | Which community-based programs (such as youth mentoring and after-school programs) effectively promote positive sexual and reproductive health outcomes among males? |
|  | 3.15 | Which community-based programs (such as youth mentoring programs and after-school programs) can help improve sexual and reproductive health outcomes among the LGBTQIA+ population? |
|  | 3.16 | How effective are current school-based sexual and reproductive health programs in improving preconception health knowledge and behaviours? |

**Domain 4: Violence and Mental Health**
Based on your knowledge and experience, rank how important each question is for further research on violence and mental health among adolescents and young adults (10-24 years) residing in Australia

Please rate each question's priority using the following scale:
High Priority (1-3): Urgent implementation needed; critical for immediate healthcare improvement
Moderate Priority (4-6): Important but less time-sensitive; should be addressed in the medium term
Low Priority (7-9): Beneficial but not urgent; can be addressed after higher priorities

**Trigger Warning!**
Some questions or topics in this domain relate to sensitive issues. These may be distressing for individuals with past experiences of trauma. If you find any section uncomfortable, you are welcome to skip that part or choose "Prefer not to answer." Your well-being comes first.

| Domain 4: Violence and Mental Health | 4.1 | What are the long-term health impacts of violence (be it domestic, sexual, or dating violence) during preconception on the health of young mothers (during or after pregnancy) and their children? |
| --- | --- | --- |
|  | 4.2 | What is the impact of mental health issues during preconception on the health of young mothers (during or after pregnancy) and their children? |
|  | 4.3 | How does the use of anti-depressants and anti-anxiety medications during the preconception period impact the health of mothers (during or after pregnancy) and their children? |
|  | 4.4 | How can the detection and treatment of mental health disorders at preconception help improve pregnancy and child health outcomes? |
|  | 4.5 | How can mental health screening and support be effectively integrated into routine preconception care for adolescents and young adults? |
|  | 4.6 | How can community health workers and groups effectively and feasibly deliver a preventive mental health intervention package, and connect young people to primary health care when needed? |
|  | 4.7 | What strategies could be developed that are effective in preventing intimate partner violence and coerced/forced sex among adolescents and young adults? |
|  | 4.8 | How do programs designed to prevent and reduce gender-based violence equip adolescents and young adults to address and reduce their risk? |

**Domain 5: Nutrition, Weight Management, and Physical Activity**

Based on your knowledge and experiences, please rank how important each question is for further research on Nutrition, Weight Management, and Physical Activity for improving the preconception health of adolescents and young adults aged 10-24 years residing in Australia.

Please rate each question's priority using the following scale:
High Priority (1-3): Urgent implementation needed; critical for immediate healthcare improvement
Moderate Priority (4-6): Important but less time-sensitive; should be addressed in the medium term
Low Priority (7-9): Beneficial but not urgent; can be addressed after higher priorities

| Domain 5: Nutrition, Weight Management, and Physical Activity | 5.1 | What is the impact of preconception (before pregnancy) weight during adolescence on the health of mothers (during or after pregnancy)? |
| --- | --- | --- |
|  | 5.2 | What is the impact of preconception (before pregnancy) weight and lifestyle during adolescence on the growth and development of their children? |
|  | 5.3 | What is the impact of preconception (before pregnancy) dietary patterns and physical activity on pregnancy and birth outcomes? |
|  | 5.4 | What is the impact of preconception (before pregnancy) dietary quality during adolescence on pregnancy and birth outcomes? |
|  | 5.5 | What are the existing nutrition interventions targeted at adolescents and young adults during preconception to improve the health of mothers (during or after pregnancy) and their children? |

**Domain 6: Smoking, Tobacco, Alcohol, and Recreational Drug Use**

Based on your knowledge and experience, rank how important each question is for further research on tobacco, alcohol and recreational drug use among adolescents and young adults (10-24 years) residing in Australia.

Please rate each question's priority using the following scale:
High Priority (1-3): Urgent implementation needed; critical for immediate healthcare improvement
Moderate Priority (4-6): Important but less time-sensitive; should be addressed in the medium term
Low Priority (7-9): Beneficial but not urgent; can be addressed after higher priorities

| Domain 6: Smoking, Tobacco, Alcohol, and Substance Use | 6.1 | What are the long-term effects of preconception exposure to tobacco smoke on pregnancy and the health of future offspring/children? |
| --- | --- | --- |
|  | 6.2 | What are the long-term effects of preconception exposure to vaping on pregnancy and the health of future offspring/children? |
|  | 6.3 | What are the long-term effects of preconception exposure to alcohol on pregnancy and the health of future offspring/children? |
|  | 6.4 | What are the long-term effects of preconception exposure to recreational drug use on pregnancy and the health of future offspring/children? |
|  | 6.5 | What is the impact of preconception interventions to prevent smoking among adolescents and young adults on pregnancy outcomes? |
|  | 6.6 | What is the impact of preconception interventions to prevent vaping among adolescents and young adults on pregnancy outcomes? |
|  | 6.7 | What is the impact of preconception interventions to prevent alcohol use among adolescents and young adults and its impact on pregnancy outcomes? |
|  | 6.8 | What is the impact of preconception interventions to prevent recreational drug use among adolescents and young adults and its impact on pregnancy outcomes? |

**Domain 7: Diseases and Infections**

Based on your knowledge and experience, rank how important each question is for further research on diseases and infections among adolescents and young adults aged 10-24 years residing in Australia.

Please rate each question's priority using the following scale:
High Priority (1-3): Urgent implementation needed; critical for immediate healthcare improvement
Moderate Priority (4-6): Important but less time-sensitive; should be addressed in the medium term
Low Priority (7-9): Beneficial but not urgent; can be addressed after higher priorities

Definitions
**Communicable diseases**, also known as infectious diseases, are illnesses caused by microorganisms (like bacteria, viruses, or parasites) that can spread from one person or animal to another, either directly or indirectly.

**Non-communicable diseases (NCDs),** also known as chronic diseases, are long-term health conditions that cannot be passed from person to person. The four main types of NCDs are heart diseases, cancers, lung diseases, and diabetes

**Human Papillomavirus (HPV)** is a very common sexually transmitted infection (STI) caused by a group of viruses, some of which can cause genital warts and, in some cases, lead to certain cancers like cervical, anal, vulvar, vaginal, penile and oropharyngeal cancers. **The HPV vaccine** protects against HPV-related cancers, including cervical cancer in women and other HPV-related cancers in men, as well as genital warts.

| Domain 7: Communicable and Non-communicable Diseases (Including Infections) | 7.1 | What are the pre-pregnancy factors that can make young mothers more likely to develop diabetes (high blood sugar levels) during pregnancy? |
| --- | --- | --- |
|  | 7.2 | What are the pre-pregnancy factors that can make young mothers more likely to develop high blood pressure during pregnancy? |
|  | 7.3 | What are the pre-pregnancy factors that can make young mothers more likely to develop preeclampsia or eclampsia during pregnancy? (Note: Pre-eclampsia is a complication of pregnancy characterised by high blood pressure and signs of damage to various organs) |
|  | 7.4 | How does the use of medications for conditions like epilepsy/seizures or heart problems during the preconception period affect the health of mothers and their children? |
|  | 7.5 | What are effective, affordable, and feasible ways to screen for long-term health conditions, such as diabetes, high blood pressure, or asthma, in adolescents and young adults at preconception? |
|  | 7.6 | What are effective, affordable, and feasible means to screen for infections among adolescents and young adults before getting pregnant? |
|  | 7.7 | What strategies are available for the prevention and management of sexually transmitted infections (STIs) and human immunodeficiency virus (HIV) as part of preconception care? |
|  | 7.8 | What is the impact of Human Papillomavirus (HPV) vaccination during adolescence on the health of mothers and their children? |

**Domain 8:  Exposure to Environment and Chemicals**
*The environment and chemical exposures may include air pollution, exposure to pesticides, radiation, passive smoking, video display terminals, anaesthetic gases, antineoplastic drugs, physical stress, and exposure to lead, selenium, and inorganic mercury.* 

Based on your knowledge and experience, rank how important each question is for further research on environment and chemical exposure (before getting pregnant) among adolescents and young adults aged 10-24 years residing in Australia. 

Please rate each question's priority using the following scale:
High Priority (1-3): Urgent implementation needed; critical for immediate healthcare improvement
Moderate Priority (4-6): Important but less time-sensitive; should be addressed in the medium term
Low Priority (7-9): Beneficial but not urgent; can be addressed after higher priorities

| Domain 8: Exposure to Environment and Chemicals | 8.1 | What is the impact of environmental and chemical exposures during preconception on the health of mothers (during or after pregnancy) and their children? |
| --- | --- | --- |
|  | 8.2 | What is the impact of preconception occupational exposures to cleaning products and disinfectants on the health of mothers (during or after pregnancy) and their children? |
|  | 8.3 | What public health policies and strategies are in place to regulate and reduce environmental exposures and harmful chemicals? |
|  | 8.4 | What strategies or approaches can help modify individuals' behaviour to reduce environmental exposure to prevent long-term health impacts? |

**Domain 9: Genetics**

Based on your knowledge and experience, rank how important each question is for further research on genetics. All questions refer to the preconception health of adolescents and young adults aged 10-24 years residing in Australia.

Please rate each question's priority using the following scale:
High Priority (1-3): Urgent implementation needed; critical for immediate healthcare improvement
Moderate Priority (4-6): Important but less time-sensitive; should be addressed in the medium term
Low Priority (7-9): Beneficial but not urgent; can be addressed after higher priorities

Definitions
**Genetic risk factors** are inherited predispositions (passed from parent to child through genes), influenced by specific variations in our genes, that increase the likelihood of developing certain diseases or conditions

**Genetic screening tests** identify individuals at risk of having or developing a genetic disorder or carrying a genetic variant for a particular disorder. It's a process of testing a population to identify those who might have a genetic condition or the potential to pass it on.

**Genetic counselling** is a communication process that helps individuals and families understand and adapt to the medical, psychological, and familial implications of genetic conditions, offering information, support, and guidance to make informed decisions.

**Genetic testing** examines a person's DNA to identify changes in genes, chromosomes, or proteins that can cause or increase the risk of certain diseases or conditions. It can be used for diagnosis, predicting risks, or guiding treatment decisions

| Domain 9: Genetics | 9.1 | What are the preconception genetic risk factors among adolescents and young adults that can affect pregnancy and child health outcomes? |
| --- | --- | --- |
|  | 9.2 | What is the role of genetic testing during preconception and its impact on pregnancy and child health outcomes? |
|  | 9.3 | How aware are adolescents and young adults of genetic screening options and genetic counselling, and how accessible are these services to them, especially in relation to preconception health? |
|  | 9.4 | What is the feasibility and cost-effectiveness of implementing community-based genetic screening programs? |

**Domain 10: At-Risk Populations**

Based on your knowledge and experience, rank how important each question is for further research. All questions refer to the preconception health of adolescents and young adults aged 10-24 years residing in Australia.
Please rate each question's priority using the following scale:
High Priority (1-3): Urgent implementation needed; critical for immediate healthcare improvement
Moderate Priority (4-6): Important but less time-sensitive; should be addressed in the medium term
Low Priority (7-9): Beneficial but not urgent; can be addressed after higher priorities

Definitions
**LGBTQIA+:** The **LGBTQIA+** acronym stands for Lesbian, Gay, Bisexual, Transgender, Queer, Intersex, Asexual, and the "+" represents other identities not explicitly mentioned within the acronym, encompassing a broad spectrum of sexual orientations, gender identities, and sex characteristics.

| Domain 10: Priority Populations | 10.1 | What are the specific preconception health needs and challenges faced by young migrants and refugees/culturally and racially marginalised (CARM)? |
| --- | --- | --- |
|  | 10.2 | What are the specific preconception health needs and challenges faced by young First Nations people? |
|  | 10.3 | What are the specific preconception health needs and challenges faced by adolescents and young adults with disabilities? |
|  | 10.4 | What are the specific preconception health needs and challenges faced by young LGBTQIA+ populations? |
|  | 10.5 | How can preconception health services be better tailored to reach and engage adolescents and young adults from migrant and refugee/culturally and racially marginalised (CARM) backgrounds? |
|  | 10.6 | How can preconception health services be better tailored to reach and engage First Nations adolescents and young adults? |
|  | 10.7 | How can preconception health services be better tailored to reach and engage adolescents and young adults with a disability? |
|  | 10.8 | How can preconception health services be better tailored to reach and engage young LGBTQIA+ populations? |
